# Low FODMAP Diet for Functional Gastrointestinal Symptoms in Quiescent Inflammatory Bowel Disease: A Systematic Review of Randomized Controlled Trials

**DOI:** 10.3390/nu12123648

**Published:** 2020-11-27

**Authors:** Maria G. Grammatikopoulou, Dimitrios G. Goulis, Konstantinos Gkiouras, Meletios P. Nigdelis, Stefanos T. Papageorgiou, Theodora Papamitsou, Alastair Forbes, Dimitrios P. Bogdanos

**Affiliations:** 1 Department of Rheumatology and Clinical Immunology, School of Health Sciences, Faculty of Medicine, University of Thessaly, Biopolis, GR-41334 Larissa, Greece; mariagram@auth.gr; 2 Unit of Reproductive Endocrinology, 1st Department of Obstetrics and Gynecology, Medical School, Faculty of Health Sciences, Aristotle University of Thessaloniki, GR-56429 Thessaloniki, Greece; dgg@auth.gr (D.G.G.); meletis.nigdelis@gmail.com (M.P.N.); 3 Faculty of Health Sciences, Medical School, Aristotle University of Thessaloniki, University Campus, GR-54124 Thessaloniki, Greece; papagesn@auth.gr; 4 Laboratory of Histology and Embryology, Faculty of Health Sciences, Medical School, Aristotle University of Thessaloniki, University Campus, GR-541249 Thessaloniki, Greece; thpapami@auth.gr; 5 Institute of Internal Medicine, University of Tartu, 51003 Tartu, Estonia; Alastair.Forbes@uea.ac.uk; 6 Division of Transplantation, Immunology and Mucosal Biology, MRC Centre for Transplantation, King’s College London Medical School, London SE5 9RS, UK

**Keywords:** ulcerative colitis, Crohn’s disease, irritable bowel syndrome, carbohydrate, nutrition therapy, gut, gastrointestinal disease, calprotectin, microbiota, gastrointestinal symptoms

## Abstract

A low FODMAP diet (LFD) has been hypothesized to relieve symptoms of functional gastrointestinal disorders (FGD) in patients with inflammatory bowel disease (IBD). The aim of the study was to systematically review the literature for randomized controlled trials (RCTs) assessing the effectiveness of the LFD in patients with IBD and FGD. Four databases were searched, but a meta-analysis was not performed due to methodological and outcomes heterogeneity. Four RCTs fulfilled the criteria, with three having some concerns in their risk of bias assessment. All interventions compared the LFDs against a “typical” or sham diet, spanning in duration from 21 days to 6 weeks. Quality of life was improved in two RCTs, while revealing inconsistent findings in the third trial, based on different assessment tools. The fecal assays revealed non-significant findings for most variables (fecal weight, pH, water content, gene count, and gut transit time) and inconsistent findings concerning stool frequency and short-chain fatty acids concentration. Levels of fecal calprotectin, CRP, or T-cell phenotype did not differ between intervention and comparator arms. Two RCTs reported a reduction in abdominal pain, while results concerning pain duration and bloating were inconsistent. In one trial, energy intake was considerably reduced among LFD participants. Regarding gut microbiota, no differences were noted. A considerable degree of methodological and outcome heterogeneity was observed, paired with results inconsistency. The available data are not sufficient to justify the claim that an LFD induces relief of FGD symptoms, although it may pave the way to a placebo response.

## 1. Introduction

The form and nutrient content of ingested food may trigger a variety of gastrointestinal (GI) symptoms through a matrix of different mechanisms, including bacterial fermentation altering gut microbiota, the induction of distinct osmotic load effects in the small bowel and colon, the production of gas in the GI tract, and the activation or suppression of immune responses [1,2]. Putative anti-inflammatory foods and elimination diets, including the low fermentable oligosaccharides, disaccharides, monosaccharides, and polyols (FODMAPs) diet (LFD), have been proposed as complementary regimes alleviating symptoms of functional gastrointestinal disorders (FGD) [3,4,5,6]. Since the LFD was designed [7], its adoption has gained traction and today, it is often recommended in clinical practice [8], with evidence synthesis indicating that adoption of the LFD reduces symptoms of irritable bowel syndrome (IBS) and functional GI symptoms (FGS) in general [9,10,11,12].

FGS, however, are also frequent among patients with inflammatory bowel diseases (IBD). It has been estimated that at least one-third of quiescent Crohn’s disease (CD) and ulcerative colitis (UC) patients experience functional intestinal problems including abdominal bloating, flatulence, pain, and changes in stool consistency and frequency unrelated to concurrent inflammation [13]. The overlap of IBD and functional gastrointestinal disorders (FGD) is often associated with lower quality of life (QoL) [14] and increased anxiety and depression [15]. Although a few studies have examined the LFD among IBD patients with FGD, the subsequent guideline recommendations advocating the adoption of an LFD as a possible treatment appear weak and of low evidence [16,17,18]. One meta-analysis [19] of two randomized controlled trials (RCTs) and an equal number of case-control studies supported use of the LFD; however, since its publication, more research has emerged, the results of which have not yet been integrated into any synthesis of evidence.

Thus, the aim of the present systematic review was to update the evidence evaluating the effectiveness of adherence to an LFD in relieving functional gastrointestinal disorders among IBD patients in remission who are experiencing FGS.

## 2. Materials and Methods

### 2.1. PICO and Research Question

The research question of the study was: What is the effect of adopting an LFD among patients with quiescent IBD who have IBS or other functional GI symptoms? The PICO criteria were: Patients with IBD in remission experiencing FGS (P); the Intervention (I) was adherence to an LFD; the Comparator (C) was a standard/sham diet, placebo, or non-FODMAP elimination diet; and the Outcome (O) was a change in FGS. The protocol was registered at the Center for Open Science (OSF) (osf.io/6qv3z) and the Preferred Reporting Items for Systematic Reviews and Meta-Analyses (PRISMA) [20] guidelines were adhered to.

### 2.2. Search Strategy

**Figure 1:**
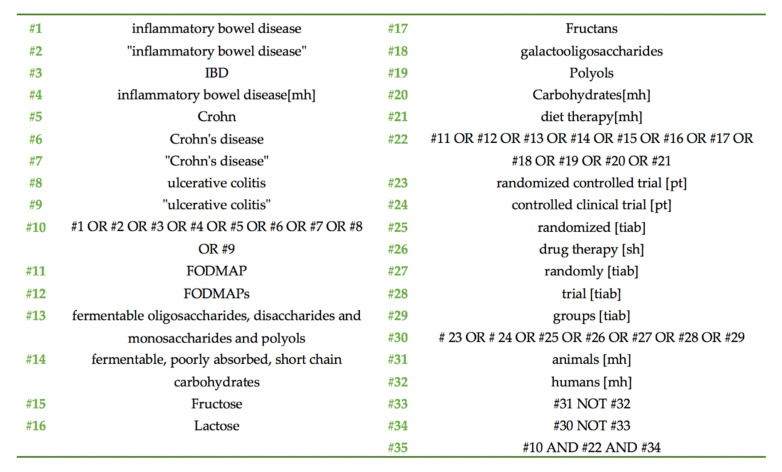
PubMed search strategy.

Three researchers (K.G., M.P.N., and M.G.G.) independently reviewed the literature systematically, searching on the PubMed (https://pubmed.ncbi.nlm.nih.gov/), Cochrane Central Register of Controlled Trials (CENTRAL; https://www.cochranelibrary.com/central), Scopus (https://www.scopus.com/home.uri), and Clinical.Trials.gov (https://www.clinicaltrials.gov/) databases for eligible randomized controlled trials (RCTs). In cases of lack of agreement, a senior researcher (D.G.G.) solved the issues through discussion and thorough appraisal of the RCTs. Search terms with a combination of MeSH terms (whenever applicable in each database) included: (inflammatory bowel disease), (IBD), (Crohn’s disease), (ulcerative colitis), and (FODMAP). The search ended in April 2020, without any restriction being imposed on the language of the retrieved trials. A detailed search strategy for PubMed is presented in Figure 1.

### 2.3. Inclusion and Exclusion Criteria

Studies were included in the qualitative synthesis when: (1) there was an RCT design (with no restriction on the randomization method or the design of the intervention arm); (2) there was a comparison of at least one treatment using a FODMAP elimination diet to a non-FODMAP elimination scheme, sham or usual diet, or placebo; (3) the study included patients with IBD in remission; (4) where an FGD was diagnosed; (5) without any restrictions concerning the age of participants or (6) the intervention duration; (7) published at any date until April 2020.

Exclusion criteria were: (1) studies without an RCT design; (2) studies performed on animals or (3) on patients with other diseases as well as IBD; (4) studies which included IBD patients who were not in remission or (5) did not have FGD; (6) studies using interventions other than a LFD; (7) studies comparing a FODMAP elimination diet to other FODMAP elimination schemes (less/more intense or different constituents); (8) studies lacking a comparator or (9) using a one group cross-over design.

### 2.4. Outcomes of Interest

All documented outcomes were considered as important, including changes in QoL, analysis of microbiota, IBD and FGD severity and symptom scores, dietary intake, and markers of immunity and inflammation.

### 2.5. Risk of Bias and Quality of the RCTs

The Cochrane Risk of Bias (RoB) 2.0 tool [21] was used to assess bias in the retrieved RCTs, with judgments falling in the categories of “low risk of bias”, “some concerns”, or “high risk of bias”. Two independent researchers (S.T.P. and K.G.) assessed RoB and a senior researcher (D.G.G.) intervened when an agreement was not reached. In parallel, the quality of the included studies was evaluated by two reviewers (M.P.N. and K.G.) using the Oxford quality score [22].

### 2.6. Data Extraction

Three researchers (M.G.G., K.G., S.T.P.) independently extracted data into predefined Excel^©^ forms. The extraction involved characteristics of the trials (e.g., origin, design, masking, ethics, funding, analysis performed), details concerning the participants (e.g., *n* in each stage, IBD diagnosis, IBS/FGD diagnosis, age), nature of the intervention (e.g., intervention, comparator, intervention duration, wash-out duration, compliance assessment, drop-outs, adverse events), and outcomes of interest.

### 2.7. Data Synthesis

A meta-analysis was intended for quantitative outcomes, where a minimum of at least three studies recording the outcome of interest would be apparent. Categorical outcomes were intended to be synthesized with odds ratios, along with the 95% confidence intervals (CI), while outcomes with continuous variables were intended to be expressed as mean differences (or standardized mean differences) with their corresponding 95% CI. A random-effects meta-analytic model was initially chosen, and statistical heterogeneity was scheduled for investigation. Results of the meta-analysis were intended to be summarized visually using forest plots, and publication bias would be assessed with funnel plots. The significance level of the meta-analysis was set at alpha = 0.05.

## 3. Results

### 3.1. Characteristics of the Trials

**Figure 2:**
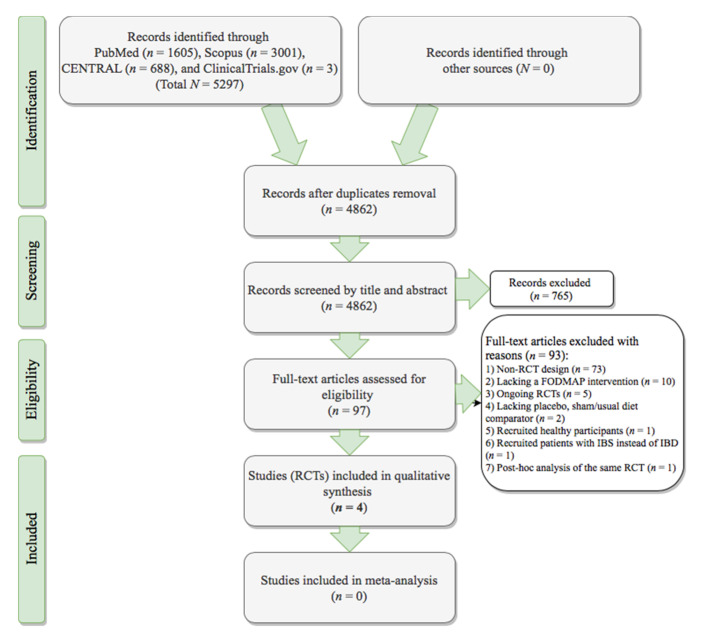
PRISMA [20] flowchart of the randomized controlled trials selection process. CENTRAL, Cochrane Central Register of Controlled Trials; IBD, inflammatory bowel disease; IBS, irritable bowel syndrome; FODMAP, Fermentable Oligo-, Di-, Monosaccharides, and Polyols; PRISMA, Preferred Reporting Items for Systematic reviews and Meta-Analyses; RCT, randomized controlled trial.

Out of 4862 records in total, four RCTs fulfilled the protocol’s criteria (Figure 2). Table 1 details the characteristics of the included trials. The Halmos et al. trial originated in Australia [23], the Cox et al. RCT was conducted in the UK [24,25], the Bodini and associates trial was Italian-based [26], and the Pedersen et al. RCT was implemented in Denmark [27,28,29]. The trial of Pedersen et al. [27,28,29] had open-label masking, and the Bodini [26] and Cox [24,25] RCTs were single-blind. The trial of Halmos et al. [23] reported only participant blinding in the manuscript text (single-blind), while referencing a previous study performed using double-blind masking [30], although the principle diagnoses of participants between the two studies failed to match. Only the Halmos et al. [23] RCT adopted a cross-over design and the remaining trials compared parallel interventions [24,25,26,27,28,29].

The number of included participants ranged from as few as 9 in the Halmos trial [23] to a total of 89 recruited by Pedersen [27,28,29] for adult patients with quiescent IBD and FGS. Halmos et al. [23] were the only investigators to limit their work to patients with CD, whereas the other studies also included some patients with UC. Cox et al. [24,25] presented the differential FGD diagnosis of all participants (functional bloating/diarrhea, mixed/unsubtyped IBS, or diarrhea-predominant IBS). Bodini and colleagues [26] reported only on patients with IBD-IBS. Petersen and associates [27,28,29] recruited patients with IBD and FGS, whereas in the Halmos et al. RCT [23], no reference to the FGD status of participants was made in the methodology, but rather, it was implied in their introduction. All participants had an FGD diagnosis based on the Rome III [32] or IV [31] criteria.

### 3.2. Intervention Characteristics

Halmos, Bodini, and Pedersen compared the LFD against a standard diet [23,26,27,28,29], whereas Cox used a sham diet of similar intensity [24,25] (Table 2). The duration of intervention lasted between 21 days in the Halmos trial [23] to a total of 6 weeks in the remaining RCTs [26,27,28,29].

The FODMAP content of each diet was reported to have been assessed through the FODMAP food composition database (Monash University, Melbourne, Australia) or via food analysis using high-performance liquid chromatography and enzymatic assays. In the Halmos trial, LFD was determined as ≤0.5 g of FODMAP per sitting [37]. Compliance was mostly assessed by dietitians [23,26,30], using food-frequency questionnaires (FFQs) [27,28,29] or food diaries [24,25,26]. Cox and associates [24,25] additionally used one question at the end of the intervention to assess adherence to the LFD “’During the 4-week trial I have followed the diet…’: never/rarely (<25% of the time), sometimes (25–50% of the time), frequently (51–75% of the time) or always (76–100% of the time)”. The Halmos trial [23] was the only one where the fiber content of the LFD was adjusted to that of the typical control diet using supplemental psyllium and resistant starch.

### 3.3. Outcomes of Interest

Disease activity was determined using a variety of tools, including the IBD Control questionnaire (IBD-Control-Q) [38], the Harvey Bradshaw Index (HBI) [33] for patients with CD, and the Partial Mayo Score PMS [36] or the Simple Clinical Colitis Activity Index (SCCAI) [39] for those with UC (Table 2).

Stool samples were collected in all four RCTs, and fecal calprotectin (fCAL) was assessed using an enzyme-linked immunosorbent assay (ELISA). In the Cox [24,25] and Halmos [40] trials, microbiome composition was also assessed using quantitative metagenomic pipeline and polymerase chain reaction (PCR) on DNA fecal samples, respectively. In parallel, different stool indices were recorded in each trial, including consistency via the Bristol Stool Form Scale (BSFS) [41], fecal water content (FWC), stool frequency, weight, pH, and short-chain fatty acid (SCFA) concentration, and gut transit time (Table 2).

Changes in the QoL of participants were assessed using the IBD-Q [34,35], the Short Form 36 (SF36) [42], the IBS Health-related QoL (HR-QoL) [43], the Short Inflammatory Bowel Disease Questionnaire (SIBDQ), and IBS-specific tools like the IBS-QoL [44] (Table 2).

Assessed inflammation markers included C-reactive protein (CRP) concentrations, which were included in all trials except that of Halmos et al. [23]. Peripheral T-cell phenotype was assessed by Cox [24,25] using fluorescently conjugated monoclonal antibodies to detect CD3, naïve (CD45RA+), effector/memory (CD45RA−), CD4, and CD8 T-cells, as well as Vδ2 unconventional T-cells. In the same RCT, gut integrin α4β7 was also examined using flow cytometry.

Changes in the dietary intake of participants following the interventions were assessed by Halmos and Cox [23,24,25], using FFQs [45] and food diaries (Table 2).

Relief of FGD symptoms was assessed using the global symptoms question (GSQ) [47,48], or 100 mm visual analog scales, assessing the severity of symptoms including bloating, abdominal pain, and wind. In the Cox RCT [24,25], flatulence was evaluated via the GI symptoms rating scale (GSRS) [46]. In the Cox and Pedersen trials [24,25,27,28,29], the IBS Severity Scoring System (IBS-SSS) [49] was applied.

### 3.4. Risk of Bias of the Included RCTs

**Figure 3:**
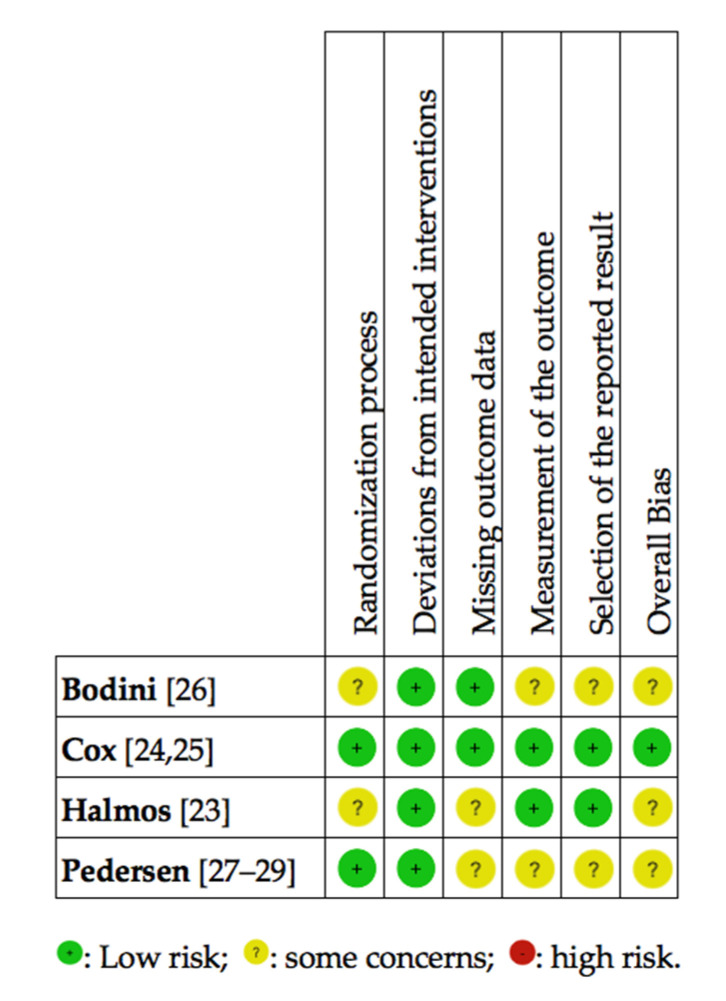
Randomized controlled trials, investigating the effects of a low FODMAP diet in patients with IBD and FGD, rated by the Cochrane risk of bias tool [21]. FGD, functional gastrointestinal disorders; FODMAP, Fermentable Oligo-, Di-, Monosaccharides, and Polyols; IBD, inflammatory bowel diseases. Note: none of the studies exhibited high risk of bias.

Summary of the risk of bias in the included RCTs revealed (Figure 3) that the trial by Cox et al. [24,25] was of low risk in all examined domains. On the other hand, the studies by Bodini et al. [26] and Halmos et al. [23] had some concerns regarding the randomization process as well as the overall bias. The Halmos et al. [23] trial also raised some concerns regarding missing outcome data. Some concerns were raised regarding missing outcome data in the trial conducted by Halmos et al. [23]. In the Bodini et al. [26] RCT, concerns were raised regarding the measurement of the outcomes and the selection of the reported findings.

### 3.5. Effect of the LFD in QoL

The Bodini, Cox, and Pedersen trials [24,25,26,27,28,29] reported an improved QoL according to IBD-specific tools following the adoption of the LFD (Table 3). Cox and colleagues [24,25] additionally reported significant improvements, specifically in the Bowel II domain score of the IBD-Q. However, the QoL, as judged by IBS-related questionnaires (IBS-QoL and SF36), failed to improve according to Pedersen [27,28,29].

### 3.6. Fecal Assays

Analyses of fecal samples in the Halmos and Cox RCTs [23,24,25] revealed inconsistent findings concerning stool frequency and SCFA concentration and a lack of differences in stool pH. Individual trials revealed that the LFD did not induce any significant effects on the FWC, fecal weight, gene count, or gut transit time [23], or in the proportion of produced stools with normal consistency [24,25]. Concerning fCAL, three trials (Halmos, Cox, and Pedersen) reported no significant effect [23,24,25,27,28,29], but Bodini et al. [26] noted a reduction following the LFD (Table 3).

### 3.7. Markers of Inflammation and Immunity

According to Bodini, Cox, and Pedersen, after commencing the LFD intervention, no difference was demonstrated in the CRP levels of participants, as compared to the controls [24,25,26,27,28,29] (Table 3). The peripheral T-cell phenotype was also largely unchanged according to Cox [24,25], with only the number of α4β7 positive Vδ2 T-cells being reduced post intervention.

### 3.8. Relief of FGD Symptoms

Findings concerning FGD symptoms were heterogeneous in terms of assessment tools and generally inconsistent when more than one trial was compared and of low evidence quality, as the majority of data points were unique to individual trials (Table 3). The severity of FGD symptoms improved in the Halmos and Pedersen trials [23,27,28,29] as measured with a VAS or the IBS-SSS, although Cox [24,25] failed to demonstrate any improvement using the latter tool. Reduction in abdominal pain was also reported by the Halmos and Pedersen RCTs [23,27,28,29] using different tools. However, when the analysis was based on a common tool—the IBS-SSS—Cox and Pedersen [24,25,27,28,29] demonstrated inconsistent findings regarding bloating symptoms and pain duration, although both showed a reduction in stool frequency and improved consistency. Finally, using the GSRS, Cox et al. [24,25] reported reduced flatulence symptoms among LFD participants at the end of the trial.

### 3.9. Changes in the Dietary Intake Following an LFD

Adherence to an LFD induced inconsistent findings in the four RCTs (Table 3). In the Cox et al. trial [24,25], equivalent energy intake between the intervention and the control group was not monitored, and the use of oral nutrient supplements (ONS) was forbidden. This resulted in reduced energy, protein, total fat, sugar, calcium, iodine, and phosphorus intake in the LFD participants. On the other hand, in the Halmos et al. trial [23], participants were instructed to eat according to their appetite to fulfill all Australian food group-specific recommendations, with their patients demonstrating an average daily energy intake of approximately 8 MJ (1900 kcal). In parallel, fiber intake was increased in the LFD group through the provision of ONS. These factors synergistically reduced possible adverse LFD effects concerning the nutrient/food group adequacy of participants, as with the exception of dietary starch, analysis of quantiles of food groups intake and comparison to the Recommended Daily Allowances failed to record differences in the intake between the LFD and control groups [23].

### 3.10. Effects on Gut Microbiota

Table 4 details all changes in fecal bacteria abundance following the LFD intervention compared with the control diet. No differences were noted in the total species abundance (relative or absolute) or the total *Bifidobacteria* sp. in either trial where this was studied [23,24,25]. Cox et al. [24,25] showed no differences in the gene count, phyla distribution, *α* and *β*-diversity, nor in targeted *Bifidobacteria* including *animalis*, *bifidum*, *breve*, and *pseudocatenulatum* species. On the other hand, a reduction in the relative abundance of *Bifidobacterium adolescentis*, *dentium*, and *longum* was recorded in the LFD group compared with the controls [24,25]. The Halmos and Cox RCTs [23,24,25] were unable to reach a unanimous finding concerning the total *Faecalibacterium prausnitzii* abundance post LFD intervention. Finally, Halmos and associates [23] suggested a reduction in the absolute and relative fecal content of *Clostridium cluster XIVa*, *Akkermansia muciniphila*, and the relative abundance of *Ruminococcus torques* following the LFD, and a lack of significant difference concerning *Roseburia*, *Lactobacilli* sp., *Ruminococcus gnavus*, and *Clostridium cluster IV*.

### 3.11. Data Synthesis

Considering the methodological and clinical heterogeneity observed in the individual studies, a meaningful meta-analysis with respect to any quantitative outcome from at least three RCTs was not considered feasible, as it would have resulted in unreasonably excessive variation in any attempt to synthesize the data.

### 3.12. Research in the Pipeline

Table 5 details all ongoing RCTs registered in Clinicaltrials.gov, examining the efficacy of the LFD on patients with IBD. Three trials in total, implemented in Mexico (NCT04143633), Iran (NCT03644602), and Denmark (NCT02469220), have results pending to be published. All trials have both subjective and objective outcomes, with intervention duration ranging between 4 and 10 weeks.

## 4. Discussion

Careful inspection revealed that the RCTs were highly heterogeneous in terms of design, participants, and outcomes. Accordingly, any attempt to recommend adherence to the LFD for relief from FGS in patients with IBD will be based on inadequate evidence. The four retrieved RCTs yielded inconsistent findings concerning all outcome domains, including disease severity, QoL, FGS relief, gut microbiota, nutrient intake, immunity and inflammation markers, stool characteristics, and fecal composition.

According to the included RCTs, conflicting results were produced concerning disease severity and FGS relief following adherence to the LFD. The pathophysiology of IBD is well understood, with the presence of chronic mucosal inflammation paired with a dysregulated immune response due to type 1 T-helper cells (in CD) or type 2 T-helper cell (in UC), resulting in partial loss of bowel integrity, while impeding epithelial barrier regeneration [50]. In FGD and IBS in particular, a persistent immune activation is apparent; therefore, even in remission, patients with IBD-IBS demonstrate dysbiosis [51], chronic relapse phases [52], a compromised immune response, an increased gut permeability, and a discorded brain-gut axis, all of which are augmented as compared to patients with IBD alone. With an overlap in signs, symptoms, pathophysiology and genetic profile in most cases, the two groups of conditions (the IBDs and the FGDs) are sometimes even regarded as the evolution of a similar disease [53]. However, even though the LFD is currently appraised as a possible treatment for IBS, the differences might well be too great for any useful extrapolation to patients with IBD-IBS who tend to exhibit more severe clinical pathophysiology with many more objective phenomena and who perhaps develop surges of the FBD elements during periods of increased stress and anxiety [15].

According to the literature, FODMAP intake has a direct effect on the gut microbiota, and adherence to an LFD reduces luminal *bifidobacteria* [10,54,55], resulting in the loss of prebiosis [56,57]. As far as patients with IBS are concerned, they tend to exhibit a greater degree of instability and a diminished microbiota diversity, both of which remain unchanged following adherence to an LFD [54,58,59]. In the present review, a lack of agreement was noted between the Halmos and Cox RCTs [23,24,25] concerning the effect of LFD on targeted bacteria. A difference in the severity of pathophysiology among the recruited participants might, in part, explain this result. Cox and associates [24,25] used patients with IBD and mixed FGD diagnoses, whereas Halmos et al. [23] selected CD patients with FGS, although detailed characteristics of the symptoms or FGD diagnosis were not provided, other than in reference to a previous article using patients with IBS only [30]. Undoubtedly, the two samples were highly heterogeneous, which might have induced different responses to the LFD. In parallel, according to Simrén [60], it is not the altered gut microbiota that induces FGS relief following LFD adherence, but rather a synergy of the FODMAP composition of the meals and the diet-microbiota produced interactions. This is why LFD studies often fail to produce significant differences in the gut microbiota from the control groups, and why GI symptoms often reappear immediately after discontinuation of the LFD [60].

Conflicting findings were also noted concerning the QoL of patients after adhering to the LFD. Considering that the IBD diet is already restrictive to some degree, and that restrictive diets can sometimes be stressful for patients with chronic disease, any effort to eliminate more foods, or impose further dietary restrictions might hamper the adherence rate, produce opposite results, and have a negative effect on the health and QoL of patients [61]. In the LFD, in particular, available dietary choices are restricted to a great degree, reducing long-term adherence [59,62]. Ooi [63] and Halmos [40] noted that extensive or inappropriate use of the LFD could have a negative impact on the health of patients. On the other hand, the duration of most LFD trials is limited and cannot ensure long-term efficacy comparable to the drug trials [64].

In parallel, due to its restrictive nature, long-term adherence to the LFD might compromise nutrient status [56,65], posing an additional risk for malnutrition among patients with IBD. Although most IBD diets fail to provide adequate amounts of all nutrients [66], the LFD in particular results in reduced fiber consumption. Among the RCTs included herein, Cox et al. [24,25] demonstrated a non-significant difference in fiber consumption, paired with a reduced energy intake in the LFD group, averaging 1697 kcal/day as compared to 1918 kcal/day demonstrated by the controls at the end of the trial. This energy intake amount is relatively low, and possibly indicative of a great proportion of low-energy reporters in the sample and/or diet records of low accuracy. Considering that in dietary analysis, the intake of all nutrients is depended on the recorded energy intake, low accuracy in the energy records might inevitably lead to inaccurate fiber consumption estimation. On the other hand, Halmos [23] accounted for the low fiber content of the LFD in advance, providing all participants with ONS, which resulted in a non-significant fiber consumption between the two groups. The reported low fiber intake when adhering to the LFD and possibly, lower energy consumption, is an important note to consider, especially in patients with IBD. The latter often fluctuate on the verge of under-nutrition and nutritional deficiencies [56], with the majority failing to receive dietetics consultation related to IBD [67]. Other concerns raised include the possible low vitamin D levels as a result of reduced dairy intake, although this was not demonstrated in the present review [59]. To ensure nutritional adequacy, Halmos [40] suggested a more gentle LFD as compared to patients with IBS alone; however, the effects of less restrictive LFDs on reducing FGD symptoms have never actually been assessed. The FODMAP inventors recommend a FODMAP-reintroduction diet in intervals lasting for two months to ensure nutrient adequacy [7]. Nevertheless, a follow-up of patients with IBD and IBS revealed that compliance was a difficult task, with only a third adhering to the LFD for more than 18 months, indicating that long-term elimination diets might be difficult to follow in the long run [68].

Undoubtedly, all nutrition interventions in IBD carry a variety of limitations. In nutrition research, expectations concerning the consumption of particular foods, personal beliefs, prior dietary advice received, dietitian reassurance, and sensory preferences, all act synergistically in creating a placebo response [69]. On the other hand, in IBS research, high rates of both placebo and nocebo responses have been suggested to occur [70]. Although in IBD alone, objective outcomes can be evaluated to limit this placebo response phenomenon [71], in the case of IBD-IBS, the placebo effect appears to be quite profound. This is augmented by a possible unmasking of the diet therapy, the discordant brain-gut axis exhibited in both IBD and FGD, and the fact that FGD severity is greatly dependent on the stress levels [72,73] and underlying biopsychosocial pathogenesis [31]. Therefore, adhering to a diet regime that is considered “healthy” might reduce anxiety and subsequently, alleviate IBS symptoms, creating a placebo response.

In parallel, examining the symptomatic effects of a diet entails substantial challenges in terms of both trial design and implementation [64]. This is why the use of subjective measures evaluating symptom severity often leads to great inconsistency in the interpretation of IBS symptoms [74]. To correct this, composite outcome scores have been proposed as optimal endpoints in IBS research [75], although these were not employed in any RCT included herein. Most RCTs failed to include objective outcomes like immune activation markers, changes in the gut microbiota or on the gut lumen [1] and interestingly, when objective outcomes were assessed, such as the CRP, fCAL, or T-cell phenotype, the lack of significant differences post-LFD adherence was apparent.

Concerns have also been raised regarding the appropriate comparator for examining the efficacy of the LFD [59]. Comparison to the usual diet, as performed in the Halmos, Bodini, and Pedersen trials [23,26,27,28,29], does not prove that the LFD is superior to the conventional IBS medical nutrition therapy (MNT). For this reason, studies comparing the LFD to other dietary treatments for IBS failed to produce significant results [76,77,78]. Therefore, by design, the existing RCTs appear to be flattering the LFD, without actually examining its effectiveness, or controlling for the subjectivity of the selected outcomes, by using composite endpoints.

## 5. Conclusions

Without a doubt, diet therapies are the most challenging to study to produce high-quality evidence [79,80]. Nevertheless, they are sought by patients and can often provide effective non-pharmacological solutions with limited adverse events. Given the known psychological effects of diet [69], it is possible that the LFD might induce a placebo response in some patients which is highly desirable. However, through careful critical appraisal of the evidence, the present systematic review failed to provide adequate evidence in terms of quality and quantity to support recommendations for an LFD for IBD patients with FGD. Publication of the results from the three ongoing RCTs is expected to add more weight to the evidence examining the efficacy of the LFD in patients with IBD and FGD. Nevertheless, as with every diet therapy, expert guidance and personalized support with FODMAP-experienced dietitians might help avoid nutritional inadequacies, while maintaining long-term adherence [81,82].

## Figures and Tables

**Table 1:** Characteristics of the included randomized controlled trials investigating the efficacy of low FODMAP diets in patients with IBD and FGS.

First Author:	Bodini [26]	Cox [24,25]	Halmos [23]	Pedersen [27,28,29]
Publication:	*Nutrition* 2019	*Gastroenterology* 2020; *J. Crohns Colitis* 2018	*Clin. Transl. Gastroenterol.* 2017	*World J. Gastroenterol.* 2017; *Dan. Med. J.* 2015; *J. Crohns Colitis* 2014
Publication type:	Full-text (*n* = 1)	Full-text (*n* = 1) and poster abstract (*n* = 1)	Full-text (*n* = 1)	Full-text (*n* = 2) and poster abstract (*n* = 1)
Study duration:	NR	2016–2017	2009–2011	2012–2013
Origin:	Italy	UK	Australia	Denmark
Registry:	-	ISRCTN17061468	ACTRN12612001185853	-
Funding:	NR	Kenneth Rainin Fndn	National Health and Medical Research Council of Australia, Eva and Les Erdi Fndn, Monash University	NR
Ethical permission:	NR	London Dulwich	Eastern Health and Monash University Human Research and Ethics Committees	Ethics Committee of Science, Denmark
RCT Design:	Parallel	Parallel	Cross-over	Parallel
Randomization:	PC-generated sequence	Block, with a 1:1 ratio, stratified by diagnosis (CD/UC) and fCAL at screening	PC-generated order	A person not involved in the study generated the random sequence and numbered the envelopes
Masking:	Single-blind (clinician)	Single-blind (patients). The terms “fermentable carbohydrates”, “low FODMAP diet”, or the diet’s mechanisms were not mentioned to participants	Double-blind (?) ^§^	Open-label
Multicenter:	-	√	-	-
Recruitment site:	Ospedale Policlinico San Martino—IRCCS per l’Oncologia, Genoa	Two large gastroenterology clinics in London	Gastroenterology clinics and the internet	Tertiary hospital in Copenhagen
Participants:	*N* = 55 IBD-IBS patients on remission or with mild disease activity (PMS < 6 or HBi < 8)	*N* = 52 quiescent * IBD patients with FGD (IBS-D, IBS-M, IBS-U, FB, or FD), LFD naïve	*N* = 9 quiescent ^œ^ CD patients with FGS	*N* = 89 IBD patients with FGS in remission, or mild-to-moderate disease
Ethnicity:	NR	√	NR	NR
CD/UC (*n*):	35/20	26/26	9/0	28/61
Criteria for IBS:	Rome IV [31]	Rome III [32]	Rome III [32] ^š^	Rome III [32]
IBD Diagnostic criteria:	endoscopic, radiologic, and histologic evaluation	NR	NR	NR
Participant age:	45 (20–75) ^†^ years	≥18 years	35 (29–41) ^ƒ^ years	40 (20–70) ^†^ years
Intervention arm:	*n* = 26	*n* = 27	*n* = 9	*n* = 44
Control arm:	*n* = 29	*n* = 25	*n* = 9	*n* = 45
Inclusion criteria:	√	√	√	√
Exclusion criteria:	√	√	√	√
HLA-DQ2/DQ8:	NR	NR	All patients were negative	NR

CD, Crohn’s disease; CRP, C-reactive protein; fCAL, fecal calprotectin; FB, functional bloating; FD, functional diarrhea; FGD, functional gastrointestinal disorders; FGS, functional gastrointestinal symptoms; Fndn, Foundation; FODMAP, Fermentable Oligo-, Di-, Monosaccharides, and Polyols; GI, gastrointestinal; HBi, Harvey Bradshaw Index for CD [33]; IBD, inflammatory bowel diseases; IBD-Q, inflammatory bowel disease—Quality of Life [34,35]; IBS, irritable bowel syndrome; IBS-D, diarrhea-predominant IBS; IBS-M, mixed IBS subtype; IBS-U, unsubtyped IBS; IRCCS, Institute for Research, Hospitalization and Health Care; LFD, Low FODMAP diet; NR, not reported; PC, personal computer; PGA, Physician Global Assessment; PMS, Partial Mayo Score for UC [36]; UC, ulcerative colitis. √ included in the study; * Defined by PGA, stable medications, no IBD flare in the previous 6 months, fCAL < 250 µg/g, and serum CRP < 10 mg/L; ^œ^ Defined by HBi < 5; ^†^ Median, range; ^ƒ^ Median, interquartile range; ^§^ Masking was reported as double-blind through a reference to a previous protocol [30], however (a) participants did not match between the two publications [23,30], and (b) in the latter publication [23], blinding of investigators was not reported in the manuscript text; ^š^ No information on IBS/FGS was provided in the manuscript presenting the RCT’s results [23]. A reference to a previous publication [30] is provided for more information concerning the sample; however, a mismatch in the sample is evident, as in the first reference, participants included IBS patients only [30], excluding all with diagnosed IBD, whereas in the latter publication [23], IBD was the primary inclusion criterion.

**Table 2:** Interventions and outcomes of the included randomized controlled trials investigating low FODMAP diet in patients with IBD and FGS.

First Author:	Bodini [26]	Cox [24,25]	Halmos [23]	Pedersen [27,28,29]
Relief assessment:	NR	GSQ	100 mm VAS	NR
Improvement definition:	NR	Achieving a 50-point reduction in IBS-SSS	NR	Achieving a 50-point reduction in IBS-SSS
Intervention:	LFD No ONS was allowed	LFD	LFD ≤ 0.5 g per sitting [37] (three main meals and three snacks daily were delivered to patients) + small quantities of psyllium and resistant starch (daily average of 3 g psyllium and 5 g Hi-Maize 220 (National Starch and Chemical Company, Bridgewater, NJ, USA), to ensure similar fiber content	LFD
Comparator:	Standard diet	Sham exclusion diet of similar intensity, burden, and nutrient intake to the LFD	typical Australian diet	Normal diet
Assessment of dietary FODMAP intake:	Detailed meals with calculated FODMAP content (NOD)	Via FODMAP database (Monash University, Melbourne, Australia)	FODMAP content for all provided food underwent FODMAP analysis via high-performance liquid chromatography and enzymatic assays	NR
Intervention duration:	6 weeks	4 weeks	21 days (each intervention)	6 weeks
Wash-out duration:	N/A	N/A	>21 days (until the symptoms had returned to the same level as during their habitual diet)	N/A
Stool:	Sample	7-day diary and fresh stool sample at baseline	5-day samples	Sample
fCAL assay:	Quantum Blue fCAL (Buhlmann Lab)	ELISA	ELISA using a commercial kit (Buhlmann EK-Cal, Schönenbuch, Switzerland)	Home-administered collecting kit and ELISA
Compliance assessment:	Dietitian (weekly phone calls and food diaries)	With a question at the end of the trial: “During the 4-week trial I have followed the diet…”: never/rarely (<25% of the time), sometimes (25–50% of the time), frequently (51–75% of the time) or always (76–100% of the time) and with 7-day food diaries	Dietitian	FFQ [45] with the most commonly consumed high-FODMAP foods adapted to the Danish population
Dropouts (*n*):	-	*n* = 6 (2 withdrew consent, 1 became pregnant, 1 initiated steroids, and 1 antibiotics) *n* = 3 for low compliance	*n* = 1 for low LFD compliance	*n* = 11 (7 for difficulty in LFD compliance and 4 for lack of compliance with registering IBS symptoms)
Non-compliant (*n*):	NR	*n* = 3	*n* = 1 from the LFD group	*n* = 7 from the LFD group
Adverse events (*n*):	NR	*n* = 2 IBD relapse (one in each group) *n* = 1 started antibiotics unrelated to IBD *n* = 1 abdominal pain (controls) *n* = 2 flu-like symptoms and sinusitis (one in each group)		NR
Primary outcomes:	Δ in PMS, HBi, IBD-Q	Δ in IBS-SSS	Δ in fecal microbiota including total, butyrate-producing (*C. leptum, F. prausnitzii, Roseburia* spp.), traditionally prebiotic (*Lactobacilli* and *Bifidobacteria* spp.), and mucus-degrading bacteria (*A. muciniphila*, *R. gnavus, R. torques*)	Δ in HBi, SCCAI, patients reporting improvement
Secondary outcomes:	Δ in CRP levels, fCAL, anthropometry	Δ in GSRS, fecal SCFA (GLC), fecal pH (InLab, Mettler Toledo probe), CRP, BSFS, IBD-Q, HBi, PMS, IBD Control Q, fecal microbiome composition and function	fecal pH, total and specific fecal SCFA concentration, severity of GI symptoms (100 mm VAS), fecal frequency and weight, FWC, whole-gut transit time, comparison of data during interventional diets to habitual diet	Δ in IBS-SSS, QoL (HR-QoL, IBS-QoL), CRP, fCAL, SIBDQ, SF36, treatment satisfaction (VAS)
Microbiome composition:	-	Via quantitative metagenomic pipeline	PCR on DNA fecal samples	-
T-cell phenotype:	-	CD3, CD45RA+, CD45RA-, CD4, CD8, Vδ2 unconventional T-cells, integrin α4β7	-	-
Timepoints:	Baseline and end (6 weeks)	Baseline and end of trial (4 weeks)	Start and end of each intervention	Baseline and 6 weeks
Analyses:	ITT *n* = 55	ITT *n* = 52 PP *n* = 43	PP *n* = 8	ITT intervention *n* = 37 ITT controls *n* = 41
Jadad score [22]:	3	3	3	2

*A. muciniphila, Akkermansia muciniphila; B. adolescentis*, *Bifidobacterium adolescentis*; *B. longum*, *Bifidobacterium longum*; BSFS, Bristol Stool Form Scale [41]; CRP, C-reactive protein; ELISA, enzyme-linked immunosorbent assay; fCAL, fecal calprotectin; FFQ, food frequency questionnaire; *F. prausnitzii*, *Faecalibacterium prausnitzii*; FGS, Functional gastrointestinal symptoms; FODMAP, Fermentable Oligo-, Di-, Monosaccharides, and Polyols; FWC, fecal water content; GI, gastrointestinal; GLC, gas–liquid chromatography; GSRS, Gastrointestinal symptoms rating scale [46]; GSQ, Global Symptom Question [47,48]; HBi, Harvey Bradshaw Index for CD [33]; HR-QoL, IBS Health-related Quality of Life [43]; IBD, inflammatory bowel diseases; IBD-Q, inflammatory bowel disease—Quality of Life [34,35]; IBD-Control-Q, IBD Control Questionnaire [38]; IBS, irritable bowel syndrome; IBS-QoL, IBS Quality of Life [44]; IBS-SSS, IBS Severity Scoring System [49]; ITT, intention to treat; LFD, Low FODMAP diet; N/A, not applicable; NOD, not other defined; NR, not reported; ONS, oral nutrient supplements; PCR, polymerase chain reaction; PMS, Partial Mayo Score for UC [36]; PP, per protocol; *R. gnavus, Ruminococcus gnavus*; *R. torques*, *Ruminococcus torques*; SCCAI, Simple clinical colitis activity index [39]; SCFA, short-chain fatty acids; SIBDQ, Short Inflammatory Bowel Disease Questionnaire; SF36, Short-form 36 [42]; VAS, Visual analogue scale.

**Table 3:** Qualitative findings of the included RCTs investigating the low FODMAP diet compared to a sham/usual diet in patients with IBD and FGS.

Outcomes			Bodini [26]	Cox [24,25]	Halmos [23]	Pedersen [27,28,29]
Disease activity	CD	HBi	↓	NS		NS
	UC	PMS	NS	NS		
		SCCAI				↓
		IBD control score [38]		↑		
Stool analyses		Fecal Frequency		↓	NS	
		% with normal Consistency (BSFS)		NS		
		Fecal weight			NS	
		Fecal pH		NS	NS	
		FWC			NS	
		SCFA concentration		↓	NS	
		Gut transit time			NS	
		fCAL	↓	NS	NS	NS
		Gene count		NS		
		whole-gut transit time			NS	
Quality of life		IBD-Q	↑	↑		
		IBS-QoL				NS
		SIBDQ				↑
		SF36				NS
Inflammation markers		CRP	NS	NS		NS
Immunity		CD4 T-cells (*n*/%)		NS/NS		
		CD8 T-cell s (*n*/%)		NS/NS		
		α4β7 positive Vδ2 T-cells (*n*)		↓		
FGS		severity of GI symptoms (100 mm VAS)			↓	
		Bloating (100 mm VAS)			↓	
		Abdominal pain (100 mm VAS)			↓	
		Wind (100 mm VAS)			↓	
		Adequate relief (%) (GSQ)		↑		
	GSRS	Flatulence		↓		
	IBS-SSS	Total score		NS		↓
		Pain duration		NS		↓
		Bloating		↓		NS
		Stool frequency & consistency		↓		↓
Dietary intake		Energy (kcal)		↓	NS ^†^	
		Starch (g)		NS	↓	
		Protein (g)		↓	NS	
		Fat (g) (total)		↓	NS	
		Sugars (g)		↓	NS	
		Calcium (mg)		↓	NR	
		Iodine (µg)		↓	NR	
		Phosphorous (mg)		↓	NR	
		Fiber (g)		NS	NS *	

BSFS, Bristol Stool Form Scale [41]; CD, Crohn’s Disease; CRP, C-reactive Protein; fCAL, fecal Calprotectin; FGS, functional gastrointestinal symptoms; FODMAP, Fermentable Oligo-, Di-, Monosaccharides, and Polyols; FWC, fecal water content; GI, gastrointestinal; GSQ, Global Symptom Question; Gastrointestinal symptoms rating scale [46]; HBi, Harvey Bradshaw Index for CD [33]; HR-QoL, IBS Health-related Quality of Life [43]; IBD, inflammatory bowel diseases; IBD-Q, inflammatory bowel disease—Quality of life [34,35]; IBS, irritable bowel syndrome; IBS-QoL, IBS Quality of Life [44]; IBS-SSS, IBS Severity Scoring System [49]; LFD, low FODMAP diet; NR, not reported; NS, not significant between intervention groups; PMS, Partial Mayo Score for UC [36]; SCCAI, Simple clinical colitis activity index [39]; SCFA, short-chain fatty acids; SF36, short-form 36 [42]; SIBDQ, Short Inflammatory Bowel Disease Questionnaire; UC, ulcerative colitis; VAS, Visual Analogue Scale; ↓ lower in LFD group as compared to controls at the end of the trial; ↑ higher in LFD compared to controls at the end of the trial; * psyllium (3 g/day) and resistant starch (5 g Hi-Maize 220/day) were added to the LFD diet (National Starch and Chemical Company, Bridgewater, NJ, USA), respectively, to ensure that only the FODMAP content of the two diets differed; ^†^ participants were advised to eat to their appetite with daily energy intake averaging at approximately 8 MJ.

**Table 4:** Findings of RCTs concerning targeted bacterial ambulance analyses (fecal samples) after adherence to a low FODMAP diet compared to a sham/habitual diet, in patients with IBD and FGS.

*Bacteria*	Cox [24,25] *	Halmos [23] ^†^	
	Relative (% Total)	Absolute (Copies of 16S rRNA Gene/g)	Relative (% Total)
Total bacteria	NS	NS	NS
*α*-diversity	NS		
*β*-diversity	NS		
Phyla distribution	NS		
*Faecalibacterium prausnitzii*	↑	NS	NS
*Roseburia*		NS	NS
*Lactobacilli* sp.		NS	NS
*Bifidobacteria* sp.	NS	NS	NS
*Bifidobacterium adolescentis*	↓		
*Bifidobacterium longum*	↓		
*Bifidobacterium animalis*	NS		
*Bifidobacterium bifidum*	NS		
*Bifidobacterium breve*	NS		
*Bifidobacterium dentium*	↓		
*Bifidobacterium pseudocatenulatum*	NS		
*Akkermansia muciniphila*		↓	↓
*Ruminococcus gnavus*		NS	NS
*Ruminococcus torques*		NS	↑
*Clostridium* *cluster IV*		NS	NS
*Clostridium cluster XIVa*		↓	↓

FGS, functional gastrointestinal symptoms; FODMAP, Fermentable Oligo-, Di-, Monosaccharides, and Polyols; IBD, inflammatory bowel diseases; LFD, low FODMAP diet; NS, not significant; RCT, randomized controlled trial; ↓ lower in the LDF participants as compared to the controls; ↑ higher in the LFD participants as compared to the controls; * the comparator was a sham diet; ^†^ the comparator was the typical Australian diet.

**Table 5:** Registered ongoing RCTs assessing the effects of the LFD on patients with IBD.

Clinical Trial Identifier	Collaborators	Design	Intervention Duration	Sample	Intervention(s), Comparator(s)	Study Duration	Primary Outcomes	Secondary Outcomes
NCT041 43633 *	(1) Hospital General de México	Parallel, single-blind (patients) RCT	10 weeks	(1) Patients with IBS; (2) Patients with UC; (3) Healthy participants (*N* = 105 normal weight or overweight adults)	(1) LFD (55% CHO, 25% fat, 20% protein) in five meals daily (2) Standard diet (55% CHO, 25% fat, 20% protein) in five meals daily. Cruciferous vegetables, fruits, and condiments were eliminated and maintenance of a normal FODMAP content was sought	February 2018 to August 2020	Nutritional status (serum TC, TG, Ca, Alb, Fe, Hb, Ht, vitamins B_12_ and D, and Cr levels)	(1) WHOQOL-BREF; (2) FFQ; (3) Body composition (BF, LBM) and anthropometry (arm, waist, and hips perimeters); (4) Gut microbiota (stool sample PCR); (5) Blood chemistry (Glu, Cr, HDL, LDL, TC); (6) IBS-SSS; (7) Symptoms severity
NCT024 69220 *	(1) North Denmark Hospital (2) Vendsyssel Hospital	Parallel RCT with quadruple masking	8 weeks	Patients with UC and IBS (*N* = 45 adults)	(1) LFD and low-FODMAP ONS; (2) LFD and high-FODMAP ONS;	June 2015 to December 2020	IBS-SSS	(1) SF36 (2) Pain (VAS)
NCT036 44602 ^†^	(1) Shariati Hospital (2) Tehran University of Medical Sciences	Parallel, open-label RCT	4 weeks	Patients with moderate UC (*N* = 32 adults)	(1) LFD (55% CHO, 25% fat, 20% protein) in six meals daily; (2) Standard UC care including PA advice, intake of low-fat dairy and meat, intake of vegetable oils and reduced refined sugars	July 2018 to April 2023	(1) Gut microbiota (via PCR)	(1) Inflammation (NOD, via ELISA)

Alb, albumin; BMR, basal metabolic rate; BF, body fat; BW, body weight; Ca, calcium; CHO, carbohydrate; Cr, creatinine; ELISA, enzyme-linked immunosorbent assay; Fe, iron; FFQ, food frequency questionnaires; FODMAP, Fermentable Oligo-, Di-, Monosaccharides, and Polyols; Glu, glucose; Hb, hemoglobin; HDL, high-density lipoprotein; Ht, hematocrit; IBD, inflammatory bowel disease; IBS, irritable bowel syndrome; IBS-SSS, IBS Severity Scoring System [49]; LBM, lean body mass; LDL, low-density lipoprotein; LFD, low-FODMAP diet; NCT, National Clinical Trials (Clinicaltrials.gov); NOD, not other defined; ONS, oral nutrient supplement; PA, physical activity; PCR, polymerase chain reaction; RCT, randomized controlled trial; SF36, Short-form 36 [42]; TC, total cholesterol; TG, triglycerides; UC, ulcerative colitis; VAS, Visual Analog Scale; WHOQOL-BREF, World Health Organization Quality of Life questionnaire; ^†^ Recruitment status completed; * Recruitment status ongoing.
